# Intestinal Activation of Notch Signaling Induces Rapid Onset Hepatic Steatosis and Insulin Resistance

**DOI:** 10.1371/journal.pone.0020767

**Published:** 2011-06-16

**Authors:** Joanna C. Fowler, Vincent R. Zecchini, Philip H. Jones

**Affiliations:** Medical Research Council Cancer Cell Unit, Cambridge, United Kingdom; Sapienza University of Rome, Italy

## Abstract

Here we investigate the effects of expressing an activated mutant of Notch (ICD-E) in an inducible transgenic mouse model. Hepatic expression of ICD-E in adult animals has no detectable phenotype, but simultaneous induction of ICD-E in both the liver and small intestine results in hepatic steatosis, lipogranuloma formation and mild insulin resistance within 96 hours. This supports work that suggests that fatty liver disease may result from disruption of the gut-liver axis. In the intestine, ICD-E expression is known to produce a transient change in the proportion of goblet cells followed by shedding of the recombinant epithelium. We report additional intestinal transcriptional changes following ICD-E expression, finding significant transcriptional down-regulation of *rpL29* (ribosomal protein L29), which is implicated in the regulation of intestinal flora. These results provide further evidence of a gut-liver axis in the development of fatty liver disease and insulin resistance and validate a new model for future studies of hepatic steatosis.

## Introduction

Non-alcoholic fatty liver disease (NAFLD) is associated with the metabolic syndrome, a cluster of factors associated with the development of insulin resistance and atherosclerosis which has reached epidemic proportions in the developed world [1]. NAFLD includes a range of disorders, from simple steatosis which may be benign to non-alcoholic steatohepatitis (NASH), in which fatty liver is associated with inflammation [2], [3]. Patients with NASH may in turn develop liver fibrosis which may progress to liver failure [4], [5].

The pathogenesis of NAFLD is complex and multiple processes are implicated in the accumulation of hepatic lipid. These include increased levels of plasma free fatty acids, due to increased lipolysis in adipose tissue or a high fat diet; increased *de-novo* lipogenesis within the liver; suppression of Very Low Density Lipoprotein (VLDL) secretion from the liver and decreased hepatic fatty acid oxidation [6], [7]. These processes may share a final common pathway in triggering endoplasmic reticulum stress and the unfolded protein response acting via the transcription factor XBP1, driving both steatosis and insulin resistance [7].

There is increasing evidence that disruption of the gut-liver axis may be involved in the pathogenesis of fatty liver disease. Gut permeability is increased in NAFLD patients and levels of circulating bacterial derived endotoxin rise in human subjects placed on a high fat diet [8], [9]. In mice, a continuous infusion of endotoxin results in fatty liver, weight gain, and hepatic insulin resistance [10]. The microflora of the intestine may also have a role in NAFLD. Obese humans and mice have a distinctive gut microflora, which confers obesity when transferred from obese mice to germ free lean animals [11], [12]. Taken together these results support a role of disruption of the epithelial barrier and/or intestinal microflora in the development of NAFLD.

In this study we investigate the hepatic phenotype produced by transgenic activation of Notch signaling in the intestine. Notch regulates many cell fate decisions in development and in adult life [13], [14]. Signal transduction occurs when the transmembrane Notch receptor is bound by ligands, such as Jagged, expressed on adjacent cells [15]. The intracellular domain of the receptor (ICD) is cleaved from the transmembrane domain by γ secretase and translocates to the nucleus where it binds the transcription factor CBF1 (RBPJ-K), leading to the recruitment of transcriptional co-activators and the expression of Notch target genes, such as members of the hairy-enhancer of split (Hes) family of transcription factors [16], [17].

Notch signaling plays a key role specifying differentiation in the intestinal epithelium in developing and adult mice [18], [19], [20], [21]. Previously we have shown that conditional expression of an activated Notch mutant fused to Enhanced Green Fluorescent Protein (EGFP) (ICD-E) in the intestine of adult mice results in a transient increase in goblet cells on the villus, within 24 hours of Notch activation, followed by crypt apoptosis and shedding of the recombinant epithelium [20]. This conditional expression system also results in gene expression in the liver, where Notch has been shown to regulate bile duct formation in developing and early post-natal mice [22], [23], [24], [25], [26].

Here we report that whereas ICD-E expression in the adult mouse liver alone has no detectable phenotype, induction of ICD-E in the intestine *and* liver of adult mice results in fatty liver disease associated with lipogranuloma formation and insulin resistance within four days. Further, we identify transcripts altered by ICD-E expression in the intestine, including *rpL29* which has a role in innate immunity. These results provide further support for the role of the intestine in NAFLD and describe a new model of this disease.

## Results

### Transgenic expression of an activated Notch mutant

**Figure 1:**
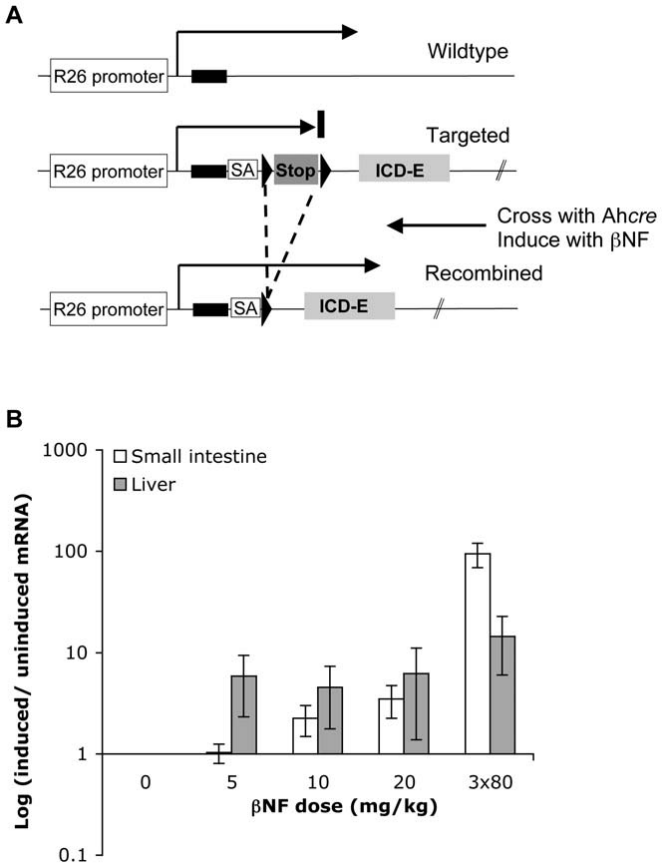
Conditional expression of an activated Notch mutant (ICD-E) in transgenic mice. A: Experimental strategy. In the R26^ICD-E/ICD-E^ transgenic strain, cDNA encoding a constitutively active Notch cytoplasmic domain mutant, fused to EGFP, is targeted to the first intron of the widely expressed Rosa26 locus. ICD-E expression is prevented by an upstream ‘STOP’ cassette, flanked by loxP sequences (triangles), which contains a transcriptional termination sequence. A splice acceptor (SA) site upstream of the ICD-E cDNA allows normal processing of the transcript after removal of the ‘STOP’ cassette. R26^ICD-E/ICD-E^ animals are crossed onto the Ah*cre* strain, in which expression of *cre* recombinase is under the control of the drug inducible *CYP1A1* promoter. Following treatment with β-napthoflavone (βNF) the ‘STOP’ cassette is excised through *cre*-mediated recombination and ICD-E is expressed [20]. B: βNF dose titration in Ah*cre*/R26^EYFP/wt^ mice. EYFP expression levels in each tissue presented as average ratio of each dose point to un-induced mice +/− s.e.m. (n = 3/dose point).

To investigate the effects of activating Notch signaling in adult mice we used a constitutively active mutant consisting of the intracellular domain of Notch1 fused to EGFP (ICD-E). This mutant localizes to the nucleus and activates transcription of a Notch responsive reporter over 100 fold when transfected into hepatocyte derived cell lines (Figure S1 and data not shown). For *in vivo* experiments we used a transgenic strain in which ICD-E was conditionally targeted to the ubiquitously expressed Rosa26 locus to generate heterozygous R26^ICD-E/wt^ mice, with a loxP flanked “STOP” cassette preventing ICD-E transcription (Figure 1A) [20]. These animals were crossed with the Ah*cre* transgenic strain to generate doubly heterozygous Ah*cre*/R26^ICD-E/wt^ animals. In these mice *cre* recombinase, under the control of the *Cyp1A1* promoter, is transiently expressed following intraperitoneal injection of the xenobiotic β–napthoflavone (βNF) [27]. This results in expression of ICD-E through *cre*-mediated removal of the “STOP” cassette. As a control we used Ah*cre*/R26^EYFP/wt^ animals which are identical to the experimental strain except enhanced yellow fluorescent protein (EYFP) rather than ICD-E is expressed from the Rosa26 locus following *cre* induction: these animals were treated with the same dose of βNF as experimental animals to control for the potential effects of transient activation of the Ah receptor [28], [29].

Treatment of the Ah*cre* strain with high doses of βNF has previously been shown to induce recombination in the liver and intestine [27]. We titrated the dose of βNF in Ah*cre*/R26^EYFP/wt^ control mice finding that a single dose of 10 mg/kg βNF results in significant *cre*-mediated gene expression in the liver with minimal induction in the small intestine or other organs whilst 3×80 mg/kg doses produce robust EYFP induction in the intestine and liver (Figure 1B). There was no EYFP induction in brown or white adipose tissue, pancreas, skeletal muscle, or brain at any dose level (data not shown).

### Transgenic activation of Notch signaling results in fatty liver within 4 days

Experimental and control mice were treated with a single 10 mg/kg dose of βNF and culled 96 hours later. Histological examination of the livers of both the experimental and control animals showed no apparent differences (Figure S2A), despite the detection of ICD-E and EYFP mRNA and protein by qRT-PCR and immunostaining respectively (data not shown). There was no increase in the transcription of *Hes1*, *Sox9* and *Hnf1β*, all of which are induced when an activated Notch mutant is expressed in hepatocytes shortly after birth [22]. In addition there were no significant differences comparing body weight, liver∶body weight ratio, percentage body fat and insulin tolerance test results in experimental and control animals (data not shown). Expression of ICD-E in adult liver alone thus results in no detectable phenotype.

**Figure 2:**
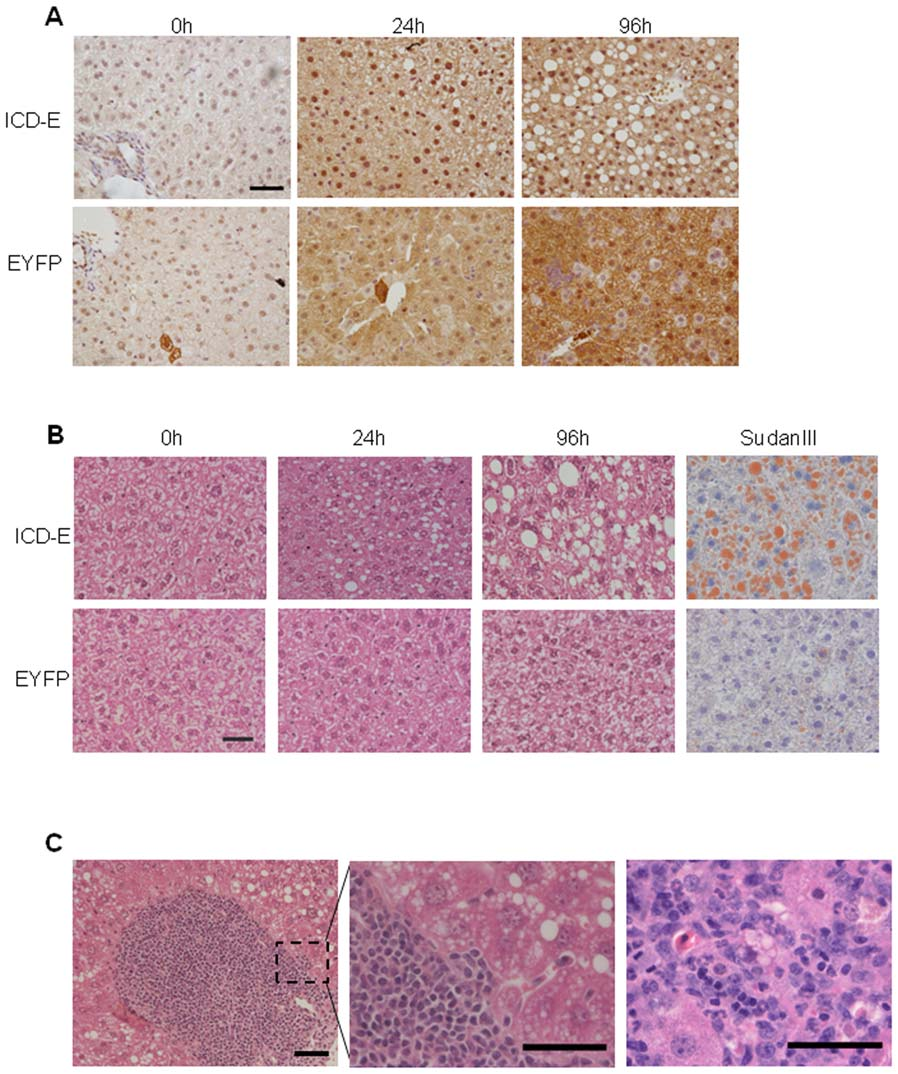
Hepatic effects of induction of ICD-E in both liver and small intestine. A: Immunohistochemical staining of liver sections from experimental (ICD-E) and control (EYFP) animals at the time points shown after induction. The anti-GFP antibody used detects both ICD-E and EYFP. Scale bar: 50 µm. B: H and E sections from time points shown in A. Right hand panels show staining for SudanIII which detects lipid. Scale bar: 50 µm. C: Two examples of lipogranulomas seen in ICD-E mice 96 hours post-induction. Scale bar (left hand panel): 100 µm. Scale bar (centre and right hand panel): 50 µm.

**Figure 3:**
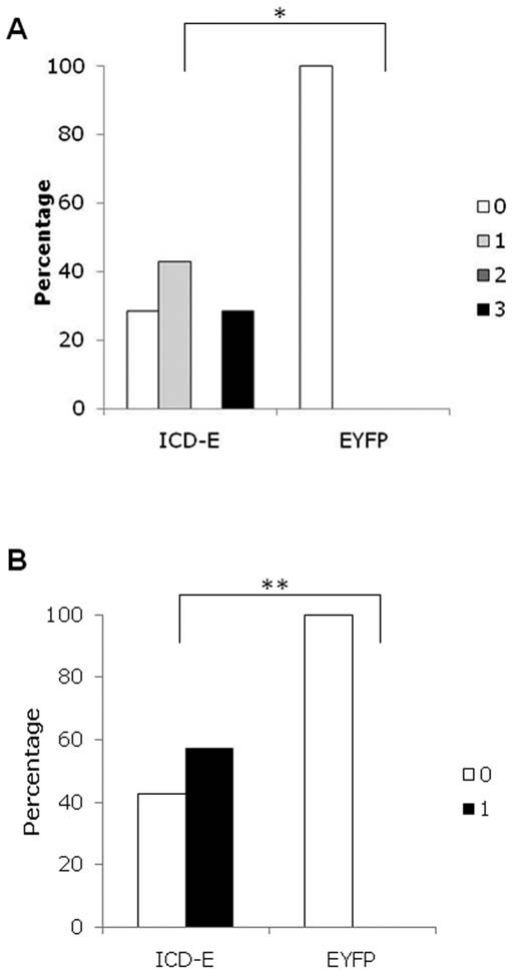
Histological grading of liver phenotype. Liver H&E sections from experimental and control induced mice were graded according to severity of steatosis (A) and presence or absence of microgranulomas (B) at 96 hours post induction. Comparison of ICD-E and EYFP by Fisher exact test *p = 0.021, **p = 0.049. n = 6 for EYFP, n = 7 for ICD-E.

We therefore investigated the effects of increasing the dose of βNF to induce gene expression in the liver and small intestine simultaneously. mRNAs encoding ICD-E or EYFP were readily detectable in both the liver and in the intestine at 24 hours post-induction (Figure S2B). In addition, immunohistochemistry revealed nuclear localized staining in the ICD-E animals and diffuse cytoplasmic staining in EYFP controls, consistent with *in vitro* results (Figure 2A, Figure S1B). Strikingly, small cytoplasmic vacuoles were visible in hepatocytes in experimental mice at 24 hours post-induction. By 96 hours the experimental livers had large fat filled vacoules, which was confirmed with the lipid stain Sudan III (Figure 2B). Areas of leukocytic infiltration consistent with lipogranuloma formation were also seen (Figure 2C). We undertook a systematic histological evaluation of the livers, scoring the severity of steatosis and lipogranuloma formation [30]. There were statistically significant differences in both steatosis grade and the occurrence of lipogranuloma in experimental animals compared with controls at 96 hours post-induction (p = 0.021 and p = 0.049 respectively by Fisher's exact test, Figure 3). Interestingly by 16 days the phenotype has almost resolved (Figure S3), this suggests that it is of rapid onset but transient in nature.

Analysis using qRT-PCR showed no difference in key transcripts from the major pathways implicated in the development of NAFLD comparing experimental and control animals (Table S1) [31], [32], [33], [34], [35], [36]. To gain further insight into the pathogenesis of NAFLD in the ICD-E mice we therefore examined liver transcription at 6 and 24 hour time points, using expression microarrays. Transcription of two genes involved in fatty acid uptake were up-regulated at 6 hours post-induction (Table 1); *Abcd2*, a gene known to be involved in the peroxisomal import of fatty acids [37], and the very low density lipoprotein receptor (*Vldlr*) [38]. It is also interesting to note that a previous report has indicated that Abcd*2* suppresses transcription of the fatty acyl chain elongase *Elovl3* and this was supported by our data at all time points [39].

**Table 1:** Hepatic transcripts found to be significantly altered on expression microarray.

Gene Symbol		Average fold change	
		6 h	24 h
**Increase**			
*Abcd2*	ATP-binding cassette, subfamily3, member 2	3.46	3.8
*ApoA4*	Apolipoprotein A4	1.53	3.31
*Cdkn1a*	Cyclin dependent kinase inhibitor	1.03	5.84
*Vldlr*	Very low density lipoprotein receptor	6.16	6.65
**Decrease**			
*Ctse*	Cathepsin E	5.52	69.97
*Elovl3*	Elongation of very long chain fatty acids like 3	5.49	3.31
*Abhd1*	Abhydrolase domain containing 1	39.53	19.26

Table shows microarray transcript fold change in experimental ICD-E mice relative to control EYFP mice at both 6 and 24 hours post-induction. Data presented as average fold change (n = 3/genotype/timepoint).

Finally we investigated the expression of known Notch target genes following ICD-E induction. *Cdkn1a*, which is directly regulated by Notch, was up-regulated in the livers of the ICD-E mice (Table 1) [40]. However hepatic transcription of another Notch target, the transcription factor *Hes1*, which has been implicated in the regulation of hepatic lipid metabolism and fatty liver disease (FLD), was unchanged (data not shown) [41]. The levels of mRNAs encoding the *Hes1* related genes *Hes5*, *Hey1* and *Hey2* were also unchanged by ICD-E expression, as were *Sox9* and *Hnf1β* (data not shown) [22], [42]. In contrast to the liver, strong induction of *Hes5* follows expression of ICD-E in the intestine [20].

### Fatty liver is associated with insulin resistance

**Figure 4:**
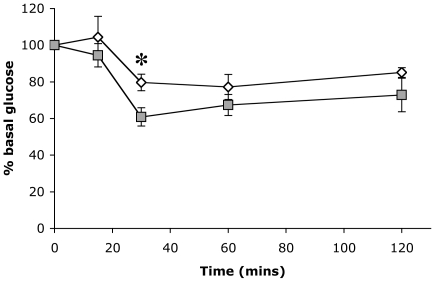
Insulin Tolerance Test at 72 hours post-induction. Mice were induced to give expression of ICD-E or EYFP in both the liver and small intestine, and an insulin tolerance test was performed 72 hours post-induction. Experimental mice (white squares, n = 7) and control mice (grey squares, n = 6). Data is presented as mean +/− s.e.m., *p = 0.018 (unpaired t-test).

Fatty liver is often associated with insulin resistance in the metabolic syndrome [43]. We therefore investigated animals with ICD-E driven FLD with an insulin tolerance test performed 72 hours post-induction (Figure 4). The glucose levels were higher in the ICD-E mice compared to EYFP controls at all time points, the difference reaching statistical significance at 30 mins post-injection (p = 0.018, unpaired t-test); indicating the ICD-E mice have developed mild insulin resistance within a very short time frame. Haematoxylin and Eosin staining of the pancreas revealed no morphological changes in the acini or islets of Langerhans (data not shown).

We measured other parameters in order to assess if there were more extensive changes in metabolism, but found there were no significant differences in total body weight, liver weight or fecal fat content between the experimental and control mice. This suggests that the phenotype was not the result of an increase in food intake or mal-absorption in the intestine (Figure S4A–E). There was also no significant difference in the weight of body fat pads when normalised to body weight between the ICD-E and EYFP mice (Figure S4F–I), indicating that substantial mobilisation of fat stores had not occurred following ICD-E induction. Finally no significant differences were seen in serum levels of alanine aminotransferase (ALT) or triglycerides: the levels of cholesterol were higher in the ICD-E mice but this was not statistically significant (Figure S5). We concluded that ICD-E induced FLD is unlikely to be due either to increased dietary fat intake or transportation from peripheral fat pads.

Next we investigated the intestinal microflora in experimental and control animals. Comparison of gut microflora between lean C57Bl/6and obese *ob/ob* mice reveals differences in the relative abundance of bacterial species, with an increase in Firmicutes and a decrease in Bacteroidetes [44]. Similar changes are found comparing obese and lean humans [45]. We assayed the relative levels of different bacterial species in induced EYFP and ICD-E mice by qRT-PCR of DNA extracted from fecal material, but found no significant difference between the experimental and control animals (Figure S6). We also attempted to quantify the levels of bacterial endotoxin in the plasma 24 hours post induction, but there were no apparent differences between the experimental and control animals (data not shown).

### Transcriptional changes following ICD-E expression in intestinal epithelium

**Figure 5:**
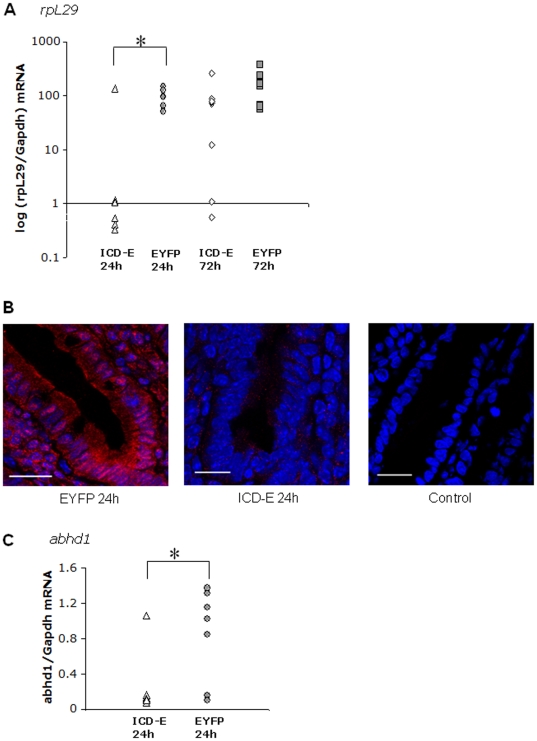
*RpL29* and *Abhd1* transcript levels are significantly down-regulated in the small intestine post-induction in ICD-E mice. A: *RpL29* transcript levels as assessed by qRT-PCR 24 hour (n = 6/genotype) and 72 hours (n = 9/genotype) post-induction. *p = 0.02, unpaired t-test. B: Immunofluorescent staining of small intestine from EYFP control (left panel) and ICD-E experimental (middle panel) mice 24 hours post induction. Sections are stained with rpL29 (red) and dapi nuclear stain (blue). The right hand panel shows no primary antibody control. Scale bar: 20 µm. C: *Abhd1* transcript levels as assessed by qRT-PCR 24 hours post-induction (n = 7/genotype) *p = 0.028, unpaired t-test.

Expression of ICD-E in the small intestine is required to trigger fatty liver. To better understand the changes in the intestinal epithelium induced by ICD-E, we assayed transcription in the small intestine by expression microarray analysis at 24 hours post induction of ICD-E or EYFP, as the intestine contains abundant recombinant epithelium at this time point [20]. Comparison of experimental and control arrays revealed significant changes in two transcripts which were validated by qRT-PCR in samples from an independent experiment. The first was the mRNA encoding ribosomal protein L29 *(rpL29)*, also known as heparin/heparan sulfate interacting protein, which was down-regulated in ICD-E animals at 24 hours post-induction (Figure 5A). This change could also be shown at the protein level (Figure 5B). *rpL29* expression recovered by 72 hours, coincident with the shedding of ICD-E expressing cells from the epithelium [20], [46]. *rpL29* has been shown to be expressed on the epithelial surface of the small intestine and exhibits broad antimicrobial activity, suggesting a role in the innate epithelial defense [47]. We also observed a significant down-regulation of the *abhd1* transcript in the intestine of ICD-E mice (Figure 5C). *Abhd1*, a member of the AB hydrolase family, is expressed in a wide range of tissues and may be involved in regulation of reactive oxygen species [48]. It is interesting to note that the expression of this gene is decreased both in the intestine and the liver, suggesting common regulation following ICD-E expression in both tissues (Table 1).

## Discussion

In the present study we demonstrate that over-expression of the activated mutant of Notch in the liver and small intestine of transgenic mice, but not in the liver alone, is sufficient to induce a phenotype that resembles human NAFLD coupled with mild insulin resistance.

There are a several environmental and genetic models of fatty liver disease including the *ob/ob* mouse strain and methionine-choline deficient diets, however all have the disadvantage of taking place over long time periods [49], [50]. The model we present develops NAFLD within 96 hours and also demonstrates that expression of transcripts from the Rosa26 locus can be specifically targeted to the liver using low doses of βNF with minimal recombination in other tissues.

**Figure 6:**
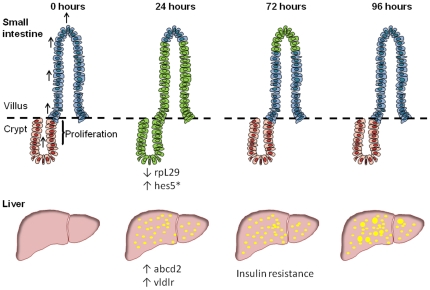
Illustration of changes following induction of ICD-E or EYFP in the liver and small intestine. Adapted from Zecchini et al (* indicates worked published by Zecchini et al [20]). In the small intestine following induction of ICD-E or EYFP by injection of β-napthoflavone recombinant epithelium (shown in green) are visible on the villus and crypt at 24 hours post induction. Over time the recombinant epithelium is shed into the intestinal lumen. In the liver fat vacuoles (shown in yellow) are apparent as early as 24 hours post induction. By 72 hours the experimental mice show signs of mild insulin resistance. Hepatic and intestinal transcriptional changes are shown below each panel.

Expression of ICD-E in the intestinal epithelium results in altered epithelial differentiation with an increased proportion of goblet cells on the villi within 24 hours, followed by a wave of apoptosis of ICD-E expressing cells in intestinal crypts [20]. The recombinant epithelium on the villus is then lost by normal cell turnover so that by 4 days the ICD-E expressing cells have been entirely replaced by wild type epithelium (Figure 6). These changes are associated with induction of *Hes1* and *Hes5*, and as shown above, down-regulation of *rpL29*. It is possible that the loss of intestinal rpL29 may contribute to the hepatic phenotype, as the protein has antimicrobial activity and is involved in the epithelial barrier against microbial invasion.

These results also illustrate the striking way in which hepatic Notch phenotypes depend on the developmental stage at which the mutation is introduced. Notch has long been linked with intra hepatic bile duct formation. Mice null for *Jagged1* and expressing a hypomorphic allele of *Notch2*, and animals null for the Notch target gene *Hes1* exhibit a severe paucity of bile ducts [24], [25], arguing Jagged/Notch2/Hes1 signaling is required for bile duct formation. Conditional deletion studies using *Albumin cre* lines have confirmed these findings [26], [51]. Expression of an *activating* Notch1 mutant in developing hepatocytes results in an increase in the density of bile ducts [23]. Strikingly, conditional expression of the same mutant in early post-natal life redirects hepatocytes into the biliary cell lineage, a fate change accompanied by induction of *Hes1*, *Sox9* and *Hnf1β* [22]. In contrast, we find no induction of these transcripts in 16 week old animals and no evidence of a bile duct phenotype, indicating Notch acts within a developmental window.

In conclusion, we show that transient, self limiting disruption of intestinal epithelium by activation of Notch signaling triggers rapid onset FLD, associated with hepatic induction of *Abcd2*, and *Vldlr*, genes associated with fatty acid uptake, mild insulin resistance and granuloma formation (Figure 6), but that this phenotype is significantly improved 16 days post induction (Figure S3). The development of FLD coincides with the loss of the antimicrobial protein rpL29 in the intestinal epithelium. These findings provide further evidence for the potential role of intestinal factors in triggering FLD.

## Materials and Methods

### Ethics Statement

All experiments were performed according to UK Government Home Office guidelines and conducted under project licence 70-6362, together with justification application Ref: CRL-NSPOR-REQ-005. The project was ethically reviewed and approved by Charles River Ethical Review Committee.

### Animals

Homozygous mice from the transgenic line Ah*cre* were crossed to homozygous R26^ICD-E/ICD-E^ mice to generate heterozygous Ah*cre*/R26^ICD-E/wt^ mice or homozygous R26^EYFP/EYFP^ animals to generate the Ah*cre*/R26^ICD-E/wt^ control strain [20], [27], [29]. All strains were maintained in a C57Bl/6 background. Control and experimental animals were approximately age matched and greater than 16 weeks old. Expression of *cre* recombinase was induced by intra peritoneal injections of β-naphthoflavone (βNF, 80 mg/kg, Sigma-Aldrich, Dorset, UK) dissolved in corn oil or DMSO at 8 mg/ml: use of corn oil or DMSO resulted in identical phenotypes in experimental animals. After induction animals were housed separately and kept on a 12 hour light/dark cycle and given ad libitum access to standard chow (VRF1(P), Special Diet Services, Essex, UK) and water.

### Animal health screens

All mice were housed in a single bio-secure isolator unit. Sentinel animals were regularly culled and serum analysed for Murine hepatitis virus, Sendai virus, Pneumonia virus of mice, Mouse minute virus, Theiler's murine encephalomyelitis (GDVII), Respiratory enteric orphan virus, Mouse rotavirus, modified Vaccinia Ankara, mouse polyoma virus, K-virus, Mouse cytomegalovirus, Mouse T-lymphotrophic virus, Mouse lymphocytic choriomeningitis virus, Hantaan virus, Mouse adenovirus and Ectromelia virus. Microbiology screening by culture was performed for *Bordatella Bronchiseptia*, *Citrobacter rodentium*, *Corynebacterium Kutscheri*, *Salmonella Sp.*, *Beta Strep Sp.*, *Streptobacillus moniliformis*, *Strep Pneumoniae*, *P. multocida*, and P. pneumotropica. PCR screening was performed for Cillia associated respiratory bacillus, *Mycoplasma pulmonis*, *H. hepaticus*, *H. bilis*, and *Helicobacter Sp.* Serology and pathological examination was performed for Tyzzer's disease. Parasitological tests were conducted for ectoparasites, endoparasites, pathogenic protozoa and serology for *E.cuniculi*. All of these tests returned negative results.

### Insulin tolerance test

The mice were fasted for 4 hours and then injected with 0.75 U/kg Humulin-S (Eli Lilly, Basingstoke, UK). Tail vein blood was collected at 0, 15, 30, 60 and 120 min intervals and glucose levels measured using an Optimum Xceed monitoring system (Abbott group, Maidenhead, Berkshire, UK).

### Fecal fat analysis

Fecal material was collected daily and snap frozen. Two pellets per animal were dried to constant weight at 65°C. Fecal fat was measured semi-quantitatively by organic extraction with chloroform∶methanol∶5 M HCl (1∶1∶2) [52]. Lipids were weighed after organic extraction and solvent evaporation and normalized to the amount of fecal material put into the extraction.

### Fecal DNA analysis

Fecal DNA was collected from snap frozen feces using a QiAmp DNA stool kit (Qiagen, Crawley UK) following manufacturer's instructions. 1 µl extracted DNA was used in subsequent qRT-PCRs.

### Clinical chemistry

Serum was collected from a terminal bleed and analysed using a Roche Diagnostics P-Module 800 clinical chemistry analyzer (Roche, Hertfordshire, UK). Triglyceride, cholesterol and ALT levels were measured using appropriate kits from Roche. All this work was carried out by Charles River Laboratories (Edinburgh, UK).

### Bacterial endotoxin quantification

Mice were induced and culled 24 hours later and plasma prepared from the terminal bleed. Endotoxin levels were quantified using the LAL gel clot assay (Associates of Cape Cod Inc., East Falmouth, MA) following the manufacturer's instructions.

### Histology

H & E sections were scored according to Kleiner et al [30]. Briefly sections were assessed for the degree of steatosis <5% parenchymal involvement was rated as 0, 5–33% (1), 34–66% (2) and >66% (3). Sections were also assessed for the presence (1) or absence (0) of microgranulomas.

### Immunohistochemistry

Antibodies used were anti-GFP (ab290 Abcam, Cambridge, UK 1∶200 dilution). Tissues were immediately removed after culling, formalin fixed and paraffin embedded. Antigen retrieval was performed by boiling under pressure in 10 mM sodium citrate pH 6 for 3 mins. Sections were treated for 30 mins in 0.3% hydrogen peroxidase at room temperature and then were blocked in 0.25% fish skin gelatin (Sigma-Aldrich) in 1×PBS for one hour. Sections were stained at 4°C overnight and visualised with the poly-HRP anti-rabbit kit (Dako). Sudan III staining was carried out according to manufacturer's instructions (Sigma-Aldrich). All sections were counterstained with haematoxylin.

Frozen sections were cut and fixed in 4% paraformaldehyde for 5 mins. These were then blocked in 0.25% fish skin gelatin in 1×PBS for an hour at room temperature and incubated with 1∶100 rpL29 (sc-103166 Santa Cruz Biotechnology Inc., Santa Cruz, CA, USA) overnight at 4°C. Sections were then incubated with 1∶500 anti-goat 546 (Invitrogen, Paisley, UK) for one hour at room temperature and nuclei were stained with Dapi.

### RNA extraction

RNA from liver and small intestine was extracted using the RNEasy columns (Qiagen) including on column DNase digestion (Qiagen). RNA from muscle was extracted using the same columns but after homogenization the sample was treated with 10 µl 10 mg/kg proteinase K at 55°C for 10 mins. For adipose tissue and the hippocampus RNA was extracted using 500 µl Qiazol (Qiagen) and homogenized using Precellys 24 (1×40 seconds 5500 rpm) (Bertin Technologies) and CK14 bead beating tubes (Cepheid UK Ltd, Stretton, UK). DNA was removed using Dnase 1 (Applied Biosystems, Warrington UK).

### qRT-PCR

qRT-PCR was performed using Taqman probes and mastermix (Applied Biosytems) according to manufacturer's instructions using a Rotorgene 3000 (Qiagen). For the primers below SYBRGreen mastermix (Qiagen) was used

GapdhF 5′-cctcgtcccgtagacaaaa-3′ Gapdh 5′-tggcaacaatctccactttg-3′

Abhd1F 5′-tactcccaagctccactgct-3′ Abhd1R 5′-aggaatcccaacatgcagac-3′

RpL29F 5′-tccgatgacatccgtgacta-3′ RpL29R 5′-tgcatcttcttcaggccttt-3′

Primers used to amplify specific bacterial groups and total bacterial 18S rRNA were taken from [53]. Standard curves were generated and the quantity of group-specific bacterial DNA calculated as a proportion of total bacterial DNA in the sample. Quantities of bacteria from the Bacteroides/Prevotella group, Bifidobacterium genus and Lactobacillus/Leuconostoc/Pediococcus group were assessed.

### Expression microarray analysis

RNA was extracted and hybridized to an Illumina MouseWG-6 bead array. All labeling, hybridization and analysis on the liver samples was performed by Expression Analysis Inc (Durham, NC) (liver samples) or by the pathology department, University of Cambridge (Cambridge, UK) (SI samples). RNA from livers from mice induced with βNF dissolved in DMSO was used. To minimize the false detection rate the liver array was analysed using PADE analysis (Permutation Analysis for Differential Expression) analysis as developed by Expression Analysis (http://www.expressionanalysis.com/docs/Illumina_Two_Group_r1.pdf) [54], [55]. For the small intestine array samples are compared by the R package limma and were filtered by an adjusted p-value which is corrected to take into account a false discovery rate based on the 0.01 p-value detection threshold [56]. All data is MIAME compliant and has been deposited in ArrayExpress (http://www.ebi.ac.uk/arrayexpress/); accession numbers E-MTAB-543 (small intestine array) and E-MTAB-544 (liver array).

### Reporter Assays

AML12 and HepA murine hepatocyte derived cell lines (ATCC, numbers CRL-2254 and CRL-1830 respectively) were cultured at 37°C and 5% CO_2_ in DMEM supplemented with 10% fetal bovine serum [57], [58]. EGFP and ICD-E cDNAs were cloned into pCS2 [20]. The Notch responsive pGa981-6 reporter plasmid, containing 12 RBP-J binding sites upstream of a minimal β–globin promoter was a gift from Ursula Juste, Munich [59]. The *renilla* luciferase plasmid pRL-TK (Promega) was used as a transfection efficiency control. Firefly and *Renilla* luciferase activity was measured with a Dual luciferase kit (Promega) 24 hours after transfection in quadruplicate wells. All results were normalised to *Renilla* luciferase activity.

## Supporting Information

Figure S1 **In vitro validation of ICD-E in hepatocyte derived cells.** A: EGFP or ICD-E was cloned into pCS2 and co-transfected with a Notch responsive firefly luciferase reporter and a renilla transfection efficiency control plasmid into the AML12 cell line. Mean firefly luciferase activity normalized to renilla expression, +/− s.d. from quadruplicate experiments is shown. B: cDNAs encoding ICD-E or EGFP were cloned into pCS2 and transfected into the hepatocyte derived AML12 cell line. Note that cells expressing ICD-E show distinct nuclear staining, whereas those transfected with EGFP show more diffuse cytoplasmic staining. Similar results were seen with the HepG2 line (data not shown).
(TIF)

Figure S2 **Persistence of ICD-E and EYFP transcripts and liver histology at lower βNF doses.** A: Liver histology in experimental and control mice where induction is targeted to the liver. B: At highest βNF dose level EYFP and ICD-E transcripts are detectable in the liver and small intestine. White represents Ah*cre* ^ICD-E/wt^ and grey represents control animals Ah*cre* ^EYFP/wt^ (n = 3 all time points). Data presented as average +/− s.e.m.
(TIF)

Figure S3 **Liver phenotype development over 16 day time course.** Liver histology in experimental and control animals after induction of ICD-E or EYFP in the liver and small intestine. Time points shown are before induction (0 hr), 24 hr, 4days and 16days post induction. n = 3/genotype/timepoint. Scale bar: 100 µm.
(TIF)

Figure S4 **Biospecimen data for ICD-E and EYFP animals.** Animals were induced to express ICD-E or EYFP in both the liver and small intestine. In all graphs white represents Ah*cre* ^ICD-E/wt^ (n = 7) and grey represents control animals Ah*cre* ^EYFP/wt^ (n = 6). There were no significant differences in any of the parameters studied. Mice were weighed daily and weight normalised to starting weight (A). Feccal pellets were collected daily and weighed (B) and data is presented as average +/− s.d. of each genotype/day. Food intake was also measured daily and is presented as the average over the whole study course (C). Fat was extracted from feccal pellets from 2 different days and quantified. Data presented as mg fat/mg feccal material (n = 3) (D). 72 hours post-induction animals were culled and the liver and all fat pads removed and weighed. Tissue weight is presented as a percentage of body weight. Liver weight (E), retroperitoneal fat pad weight (F), mesenteric fat pad weight (G), inguinal epididymal fat pad weight (H), brown fat pad weight (I).
(TIF)

Figure S5 **Clinical chemistry data for ICD-E and EYFP mice.** Mice were induced to give expression of ICD-E (white) or EYFP (grey) in both the liver and small intestine and culled 72 hours later. Serum levels of ALT (A), cholesterol (B) and triglycerides (C) were quantified (n = 6).
(TIF)

Figure S6 **Analysis of fecal DNA.** Mice were induced and fecal pellets collected 72 hours later and DNA extracted. Levels of specific groups of bacteria (Bacteroides/Prevotella (A), Lactobacillus/Leuconostoc/Pediococcus (B) and Bifidobacterium (C)) as a proportion of total bacteria DNA, were assessed by qRT-PCR in ICD-E (white) and EYFP (grey) mice. N = 6/genotype.
(TIF)

Table S1 Transcriptional changes in genes implicated in FLD. Hepatic transcriptional changes in genes associated with main pathways leading to fatty liver (i.e. increased fatty acid uptake, increased free fatty acid esterification, increased in *de novo* lipogenesis and impaired triglyceride secretion or β-oxidation) at either 24 hours or 96 hours post-induction. mRNA levels were quantified by qRT-PCR and normalized to Gapdh, and the ratio of ICD-E to EYFP calculated. Data presented as average +/− s.e.m (n = 5/genotype/timepoint, except EYFP 24 hours where n = 6).
(DOC)

## References

[1] Lloyd-Jones D, Adams R, Carnethon M, De Simone G, Ferguson TB (2009). Heart disease and stroke statistics–2009 update: a report from the American Heart Association Statistics Committee and Stroke Statistics Subcommittee.. Circulation.

[2] Ekstedt M, Franzen LE, Mathiesen UL, Thorelius L, Holmqvist M (2006). Long-term follow-up of patients with NAFLD and elevated liver enzymes.. Hepatology.

[3] Feldstein AE, Canbay A, Angulo P, Taniai M, Burgart LJ (2003). Hepatocyte apoptosis and fas expression are prominent features of human nonalcoholic steatohepatitis.. Gastroenterology.

[4] Feldstein AE, Charatcharoenwitthaya P, Treeprasertsuk S, Benson JT, Enders FB (2009). The natural history of non-alcoholic fatty liver disease in children: a follow-up study for up to 20 years.. Gut.

[5] Adams LA, Sanderson S, Lindor KD, Angulo P (2005). The histological course of nonalcoholic fatty liver disease: a longitudinal study of 103 patients with sequential liver biopsies.. J Hepatol.

[6] Donnelly KL, Smith CI, Schwarzenberg SJ, Jessurun J, Boldt MD (2005). Sources of fatty acids stored in liver and secreted via lipoproteins in patients with nonalcoholic fatty liver disease.. J Clin Invest.

[7] Savage DB, Semple RK (2010). Recent insights into fatty liver, metabolic dyslipidaemia and their links to insulin resistance.. Curr Opin Lipidol.

[8] Turnbaugh PJ, Ley RE, Mahowald MA, Magrini V, Mardis ER (2006). An obesity-associated gut microbiome with increased capacity for energy harvest.. Nature.

[9] Turnbaugh PJ, Hamady M, Yatsunenko T, Cantarel BL, Duncan A (2009). A core gut microbiome in obese and lean twins.. Nature.

[10] Amar J, Burcelin R, Ruidavets JB, Cani PD, Fauvel J (2008). Energy intake is associated with endotoxemia in apparently healthy men.. Am J Clin Nutr.

[11] Miele L, Valenza V, La Torre G, Montalto M, Cammarota G (2009). Increased intestinal permeability and tight junction alterations in nonalcoholic fatty liver disease.. Hepatology.

[12] Cani PD, Amar J, Iglesias MA, Poggi M, Knauf C (2007). Metabolic endotoxemia initiates obesity and insulin resistance.. Diabetes.

[13] Fiuza UM, Arias AM (2007). Cell and molecular biology of Notch.. J Endocrinol.

[14] Bray SJ (2006). Notch signalling: a simple pathway becomes complex.. Nat Rev Mol Cell Biol.

[15] Lindsell CE, Shawber CJ, Boulter J, Weinmaster G (1995). Jagged: a mammalian ligand that activates Notch1.. Cell.

[16] Jarriault S, Brou C, Logeat F, Schroeter EH, Kopan R (1995). Signalling downstream of activated mammalian Notch.. Nature.

[17] Furriols M, Bray S (2001). A model Notch response element detects Suppressor of Hairless-dependent molecular switch.. Curr Biol.

[18] Fre S, Huyghe M, Mourikis P, Robine S, Louvard D (2005). Notch signals control the fate of immature progenitor cells in the intestine.. Nature.

[19] van Es JH, van Gijn ME, Riccio O, van den Born M, Vooijs M (2005). Notch/gamma-secretase inhibition turns proliferative cells in intestinal crypts and adenomas into goblet cells.. Nature.

[20] Zecchini V, Domaschenz R, Winton D, Jones P (2005). Notch signaling regulates the differentiation of post-mitotic intestinal epithelial cells.. Genes Dev.

[21] Stanger BZ, Datar R, Murtaugh LC, Melton DA (2005). Direct regulation of intestinal fate by Notch.. Proc Natl Acad Sci U S A.

[22] Zong Y, Panikkar A, Xu J, Antoniou A, Raynaud P (2009). Notch signaling controls liver development by regulating biliary differentiation.. Development.

[23] Sparks EE, Huppert KA, Brown MA, Washington MK, Huppert SS (2010). Notch signaling regulates formation of the three-dimensional architecture of intrahepatic bile ducts in mice.. Hepatology.

[24] Kodama Y, Hijikata M, Kageyama R, Shimotohno K, Chiba T (2004). The role of notch signaling in the development of intrahepatic bile ducts.. Gastroenterology.

[25] McCright B, Lozier J, Gridley T (2002). A mouse model of Alagille syndrome: Notch2 as a genetic modifier of Jag1 haploinsufficiency.. Development.

[26] Geisler F, Nagl F, Mazur PK, Lee M, Zimber-Strobl U (2008). Liver-specific inactivation of Notch2, but not Notch1, compromises intrahepatic bile duct development in mice.. Hepatology.

[27] Ireland H, Kemp R, Houghton C, Howard L, Clarke AR (2004). Inducible Cre-mediated control of gene expression in the murine gastrointestinal tract: effect of loss of beta-catenin.. Gastroenterology.

[28] Lee JH, Wada T, Febbraio M, He J, Matsubara T (2010). A novel role for the dioxin receptor in fatty acid metabolism and hepatic steatosis.. Gastroenterology.

[29] Srinivas S, Watanabe T, Lin CS, William CM, Tanabe Y (2001). Cre reporter strains produced by targeted insertion of EYFP and ECFP into the ROSA26 locus.. BMC Dev Biol.

[30] Kleiner DE, Brunt EM, Van Natta M, Behling C, Contos MJ (2005). Design and validation of a histological scoring system for nonalcoholic fatty liver disease.. Hepatology.

[31] van der Vusse GJ, van Bilsen M, Glatz JF, Hasselbaink DM, Luiken JJ (2002). Critical steps in cellular fatty acid uptake and utilization.. Mol Cell Biochem.

[32] Sundaram M, Yao Z (2010). Recent progress in understanding protein and lipid factors affecting hepatic VLDL assembly and secretion.. Nutr Metab (Lond).

[33] Reddy JK, Rao MS (2006). Lipid metabolism and liver inflammation. II. Fatty liver disease and fatty acid oxidation.. Am J Physiol Gastrointest Liver Physiol.

[34] Inoue Y, Inoue J, Lambert G, Yim SH, Gonzalez FJ (2004). Disruption of hepatic C/EBPalpha results in impaired glucose tolerance and age-dependent hepatosteatosis.. J Biol Chem.

[35] Erion DM, Ignatova ID, Yonemitsu S, Nagai Y, Chatterjee P (2009). Prevention of hepatic steatosis and hepatic insulin resistance by knockdown of cAMP response element-binding protein.. Cell Metab.

[36] Dufour JF, Clavien PA (2009). Signaling pathways in liver diseases.

[37] Ferrer I, Kapfhammer JP, Hindelang C, Kemp S, Troffer-Charlier N (2005). Inactivation of the peroxisomal ABCD2 transporter in the mouse leads to late-onset ataxia involving mitochondria, Golgi and endoplasmic reticulum damage.. Hum Mol Genet.

[38] Degrace P, Moindrot B, Mohamed I, Gresti J, Du ZY (2006). Upregulation of liver VLDL receptor and FAT/CD36 expression in LDLR−/− apoB100/100 mice fed trans-10,cis-12 conjugated linoleic acid.. J Lipid Res.

[39] Brolinson A, Fourcade S, Jakobsson A, Pujol A, Jacobsson A (2008). Steroid hormones control circadian Elovl3 expression in mouse liver.. Endocrinology.

[40] Devgan V, Mammucari C, Millar SE, Brisken C, Dotto GP (2005). p21WAF1/Cip1 is a negative transcriptional regulator of Wnt4 expression downstream of Notch1 activation.. Genes Dev.

[41] Lemke U, Krones-Herzig A, Berriel Diaz M, Narvekar P, Ziegler A (2008). The glucocorticoid receptor controls hepatic dyslipidemia through Hes1.. Cell Metab.

[42] Fischer A, Gessler M (2007). Delta-Notch–and then? Protein interactions and proposed modes of repression by Hes and Hey bHLH factors.. Nucleic Acids Res.

[43] Angulo P (2002). Nonalcoholic fatty liver disease.. N Engl J Med.

[44] Ley RE, Backhed F, Turnbaugh P, Lozupone CA, Knight RD (2005). Obesity alters gut microbial ecology.. Proc Natl Acad Sci U S A.

[45] Ley RE, Turnbaugh PJ, Klein S, Gordon JI (2006). Microbial ecology: human gut microbes associated with obesity.. Nature.

[46] Stappenbeck TS, Wong MH, Saam JR, Mysorekar IU, Gordon JI (1998). Notes from some crypt watchers: regulation of renewal in the mouse intestinal epithelium.. Curr Opin Cell Biol.

[47] Meyer-Hoffert U, Hornef M, Henriques-Normark B, Normark S, Andersson M (2008). Identification of heparin/heparan sulfate interacting protein as a major broad-spectrum antimicrobial protein in lung and small intestine.. FASEB J.

[48] Stoelting M, Geyer M, Reuter S, Reichelt R, Bek MJ (2009). Alpha/beta hydrolase 1 is upregulated in D5 dopamine receptor knockout mice and reduces O2- production of NADPH oxidase.. Biochem Biophys Res Commun.

[49] Rizki G, Arnaboldi L, Gabrielli B, Yan J, Lee GS (2006). Mice fed a lipogenic methionine-choline-deficient diet develop hypermetabolism coincident with hepatic suppression of SCD-1.. J Lipid Res.

[50] Anstee QM, Goldin RD (2006). Mouse models in non-alcoholic fatty liver disease and steatohepatitis research.. Int J Exp Pathol.

[51] Loomes KM, Russo P, Ryan M, Nelson A, Underkoffler L (2007). Bile duct proliferation in liver-specific Jag1 conditional knockout mice: effects of gene dosage.. Hepatology.

[52] Nelson WR, O'Hopp SJ (1969). A semiquantitative screening test and quantitative assay for total fecal fat.. Clin Chem.

[53] Furet JP, Firmesse O, Gourmelon M, Bridonneau C, Tap J (2009). Comparative assessment of human and farm animal faecal microbiota using real-time quantitative PCR.. FEMS Microbiol Ecol.

[54] Li Y, Piatigorsky J (2009). Targeted deletion of Dicer disrupts lens morphogenesis, corneal epithelium stratification, and whole eye development.. Dev Dyn.

[55] Heidenfelder BL, Reif DM, Harkema JR, Cohen Hubal EA, Hudgens EE (2009). Comparative microarray analysis and pulmonary changes in Brown Norway rats exposed to ovalbumin and concentrated air particulates.. Toxicol Sci.

[56] Smyth GK (2004). Linear models and empirical bayes methods for assessing differential expression in microarray experiments.. Stat Appl Genet Mol Biol.

[57] Wu JC, Merlino G, Fausto N (1994). Establishment and characterization of differentiated, nontransformed hepatocyte cell lines derived from mice transgenic for transforming growth factor alpha.. Proc Natl Acad Sci U S A.

[58] Darlington GJ, Bernhard HP, Miller RA, Ruddle FH (1980). Expression of liver phenotypes in cultured mouse hepatoma cells.. J Natl Cancer Inst.

[59] Strobl LJ, Hofelmayr H, Stein C, Marschall G, Brielmeier M (1997). Both Epstein-Barr viral nuclear antigen 2 (EBNA2) and activated Notch1 transactivate genes by interacting with the cellular protein RBP-J kappa.. Immunobiology.

