# Supplementary figures and images for: Intestinal Activation of Notch Signaling Induces Rapid Onset Hepatic Steatosis and Insulin Resistance
Source: PLoS One. 2011 Jun 16;6(6):e20767. doi: 10.1371/journal.pone.0020767 (PMC3116826; doi:10.1371/journal.pone.0020767)

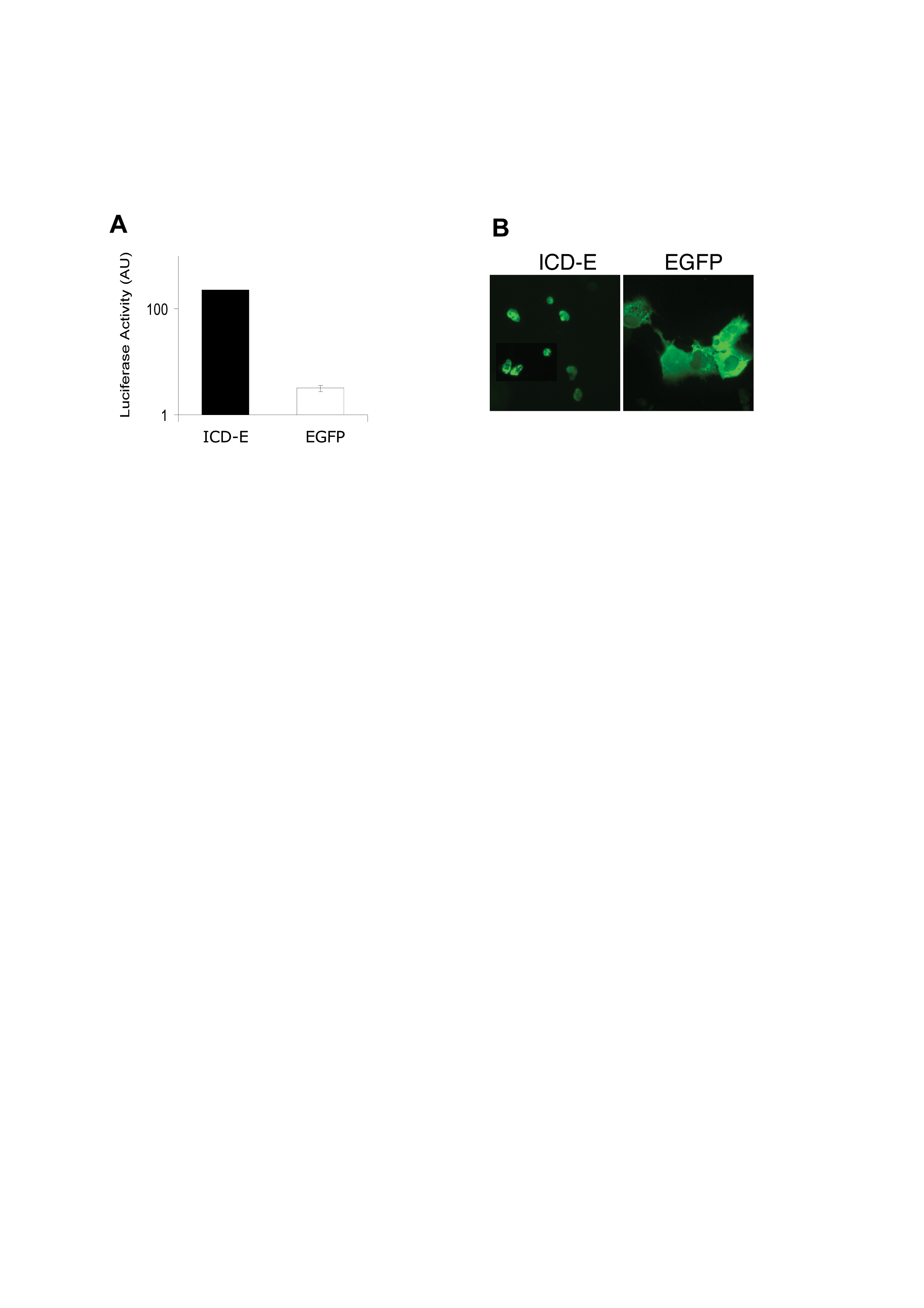

Supplement: Figure S1 — In vitro validation of ICD-E in hepatocyte derived cells. A: EGFP or ICD-E was cloned into pCS2 and co-transfected with a Notch responsive firefly luciferase reporter and a renilla transfection efficiency control plasmid into the AML12 cell line. Mean firefly luciferase activity normalized to renilla expression, +/− s.d. from quadruplicate experiments is shown. B: cDNAs encoding ICD-E or EGFP were cloned into pCS2 and transfected into the hepatocyte derived AML12 cell line. Note that cells expressing ICD-E show distinct nuclear staining, whereas those transfected with EGFP show more diffuse cytoplasmic staining. Similar results were seen with the HepG2 line (data not shown). (TIF) [file pone.0020767.s001.tif]

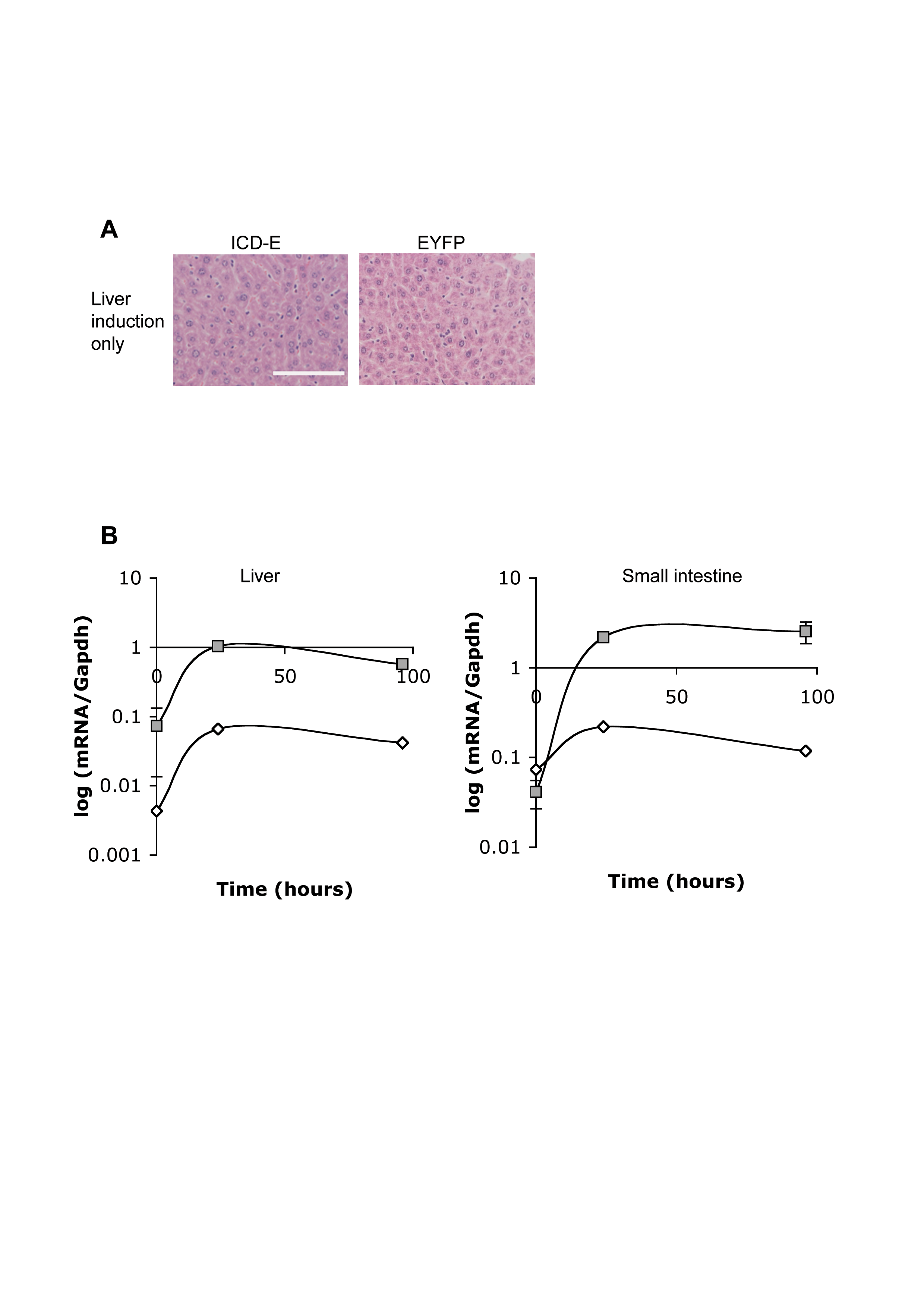

Supplement: Figure S2 — Persistence of ICD-E and EYFP transcripts and liver histology at lower βNF doses. A: Liver histology in experimental and control mice where induction is targeted to the liver. B: At highest βNF dose level EYFP and ICD-E transcripts are detectable in the liver and small intestine. White represents Ahcre ICD-E/wt and grey represents control animals Ahcre EYFP/wt (n = 3 all time points). Data presented as average +/− s.e.m. (TIF) [file pone.0020767.s002.tif]

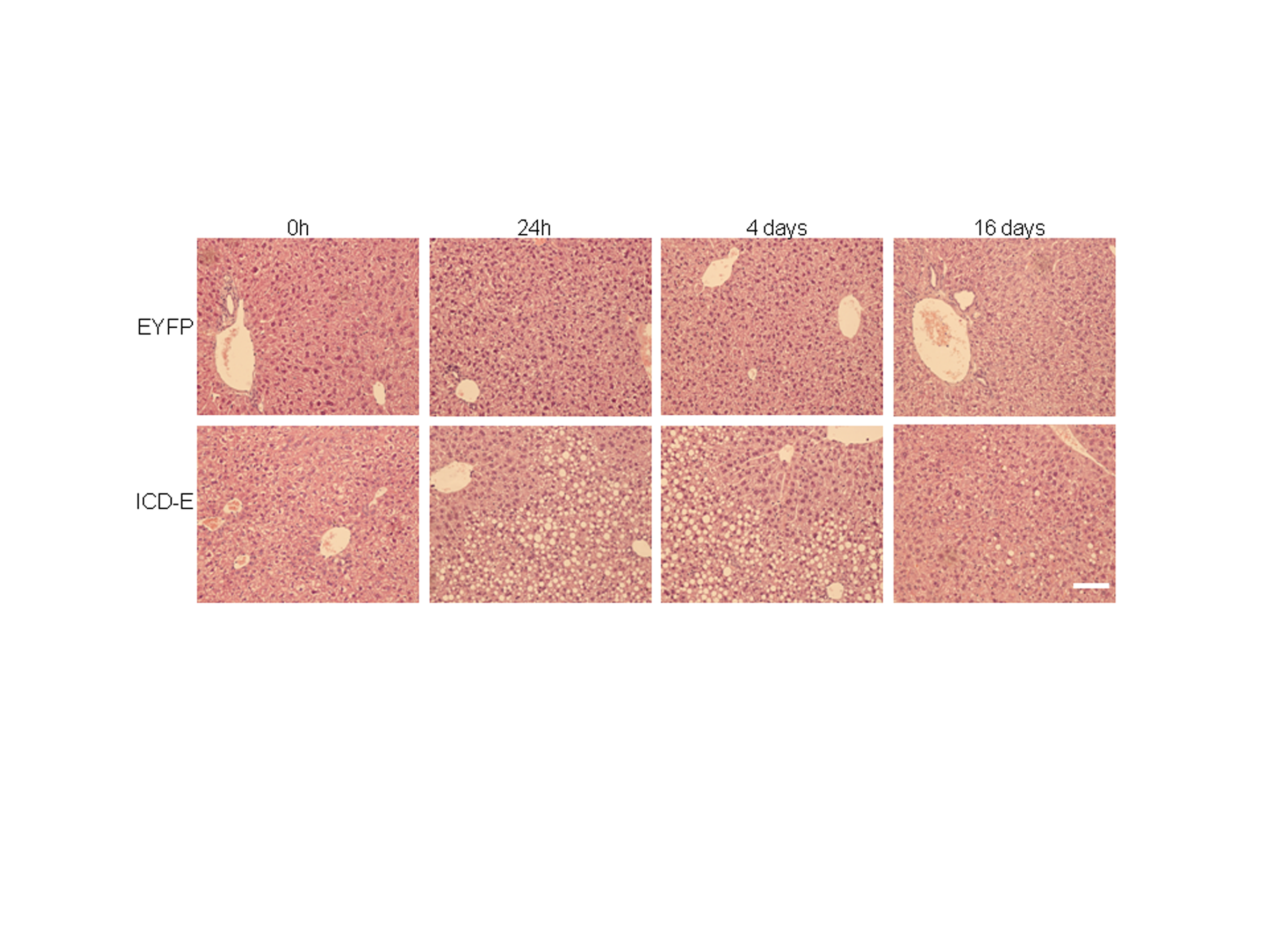

Supplement: Figure S3 — Liver phenotype development over 16 day time course. Liver histology in experimental and control animals after induction of ICD-E or EYFP in the liver and small intestine. Time points shown are before induction (0 hr), 24 hr, 4days and 16days post induction. n = 3/genotype/timepoint. Scale bar: 100 µm. (TIF) [file pone.0020767.s003.tif]

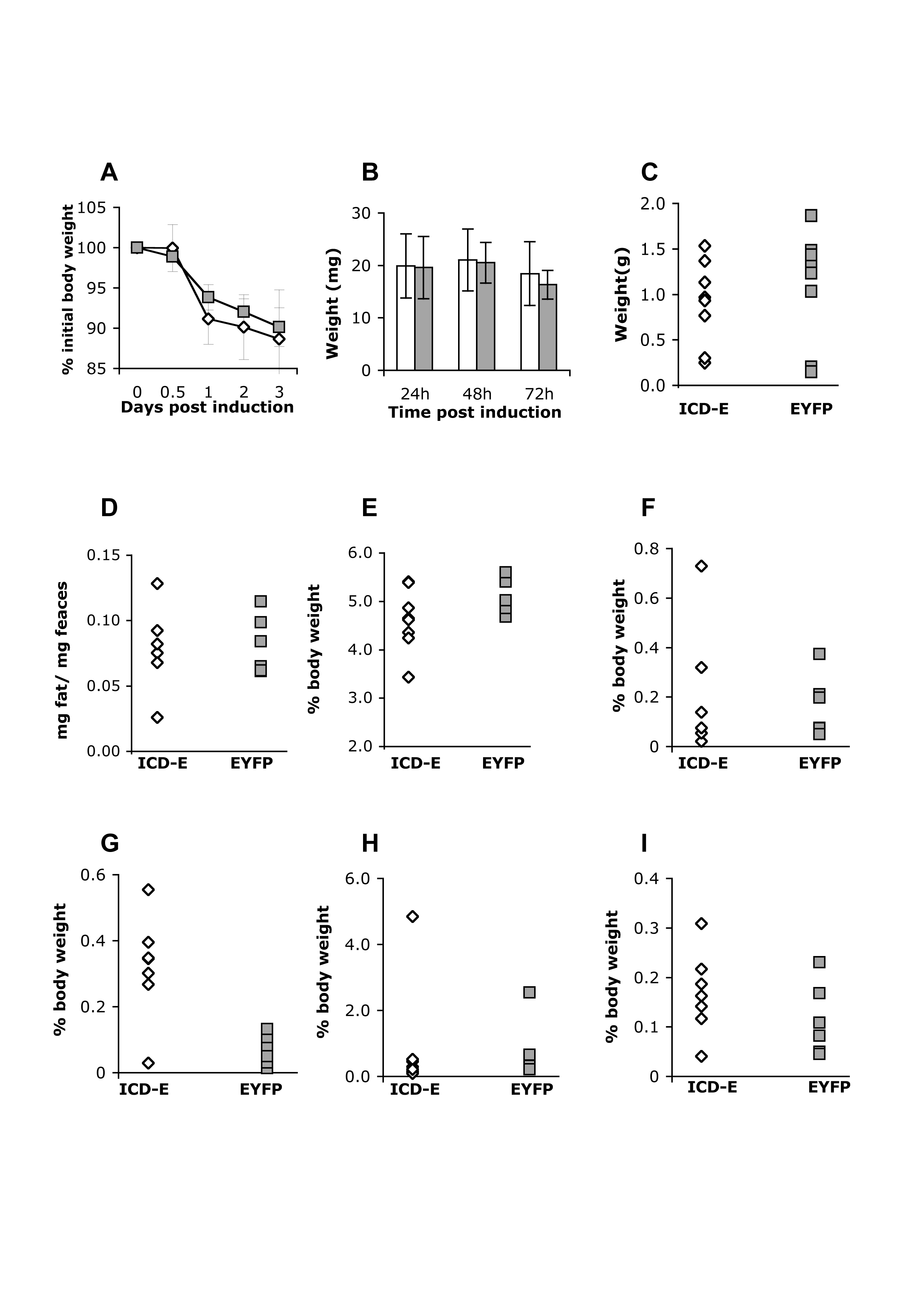

Supplement: Figure S4 — Biospecimen data for ICD-E and EYFP animals. Animals were induced to express ICD-E or EYFP in both the liver and small intestine. In all graphs white represents Ahcre ICD-E/wt (n = 7) and grey represents control animals Ahcre EYFP/wt (n = 6). There were no significant differences in any of the parameters studied. Mice were weighed daily and weight normalised to starting weight (A). Feccal pellets were collected daily and weighed (B) and data is presented as average +/− s.d. of each genotype/day. Food intake was also measured daily and is presented as the average over the whole study course (C). Fat was extracted from feccal pellets from 2 different days and quantified. Data presented as mg fat/mg feccal material (n = 3) (D). 72 hours post-induction animals were culled and the liver and all fat pads removed and weighed. Tissue weight is presented as a percentage of body weight. Liver weight (E), retroperitoneal fat pad weight (F), mesenteric fat pad weight (G), inguinal epididymal fat pad weight (H), brown fat pad weight (I). (TIF) [file pone.0020767.s004.tif]

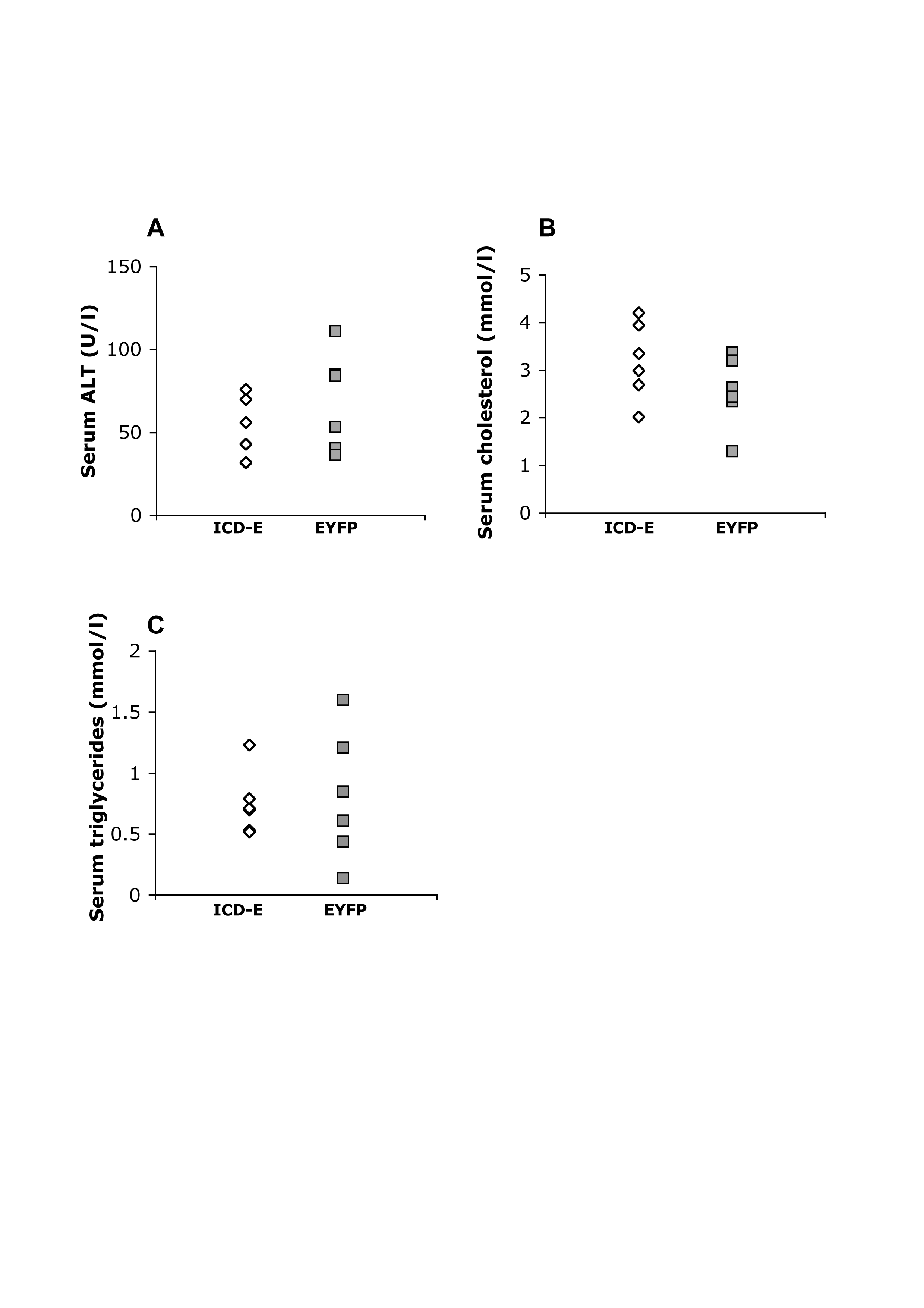

Supplement: Figure S5 — Clinical chemistry data for ICD-E and EYFP mice. Mice were induced to give expression of ICD-E (white) or EYFP (grey) in both the liver and small intestine and culled 72 hours later. Serum levels of ALT (A), cholesterol (B) and triglycerides (C) were quantified (n = 6). (TIF) [file pone.0020767.s005.tif]

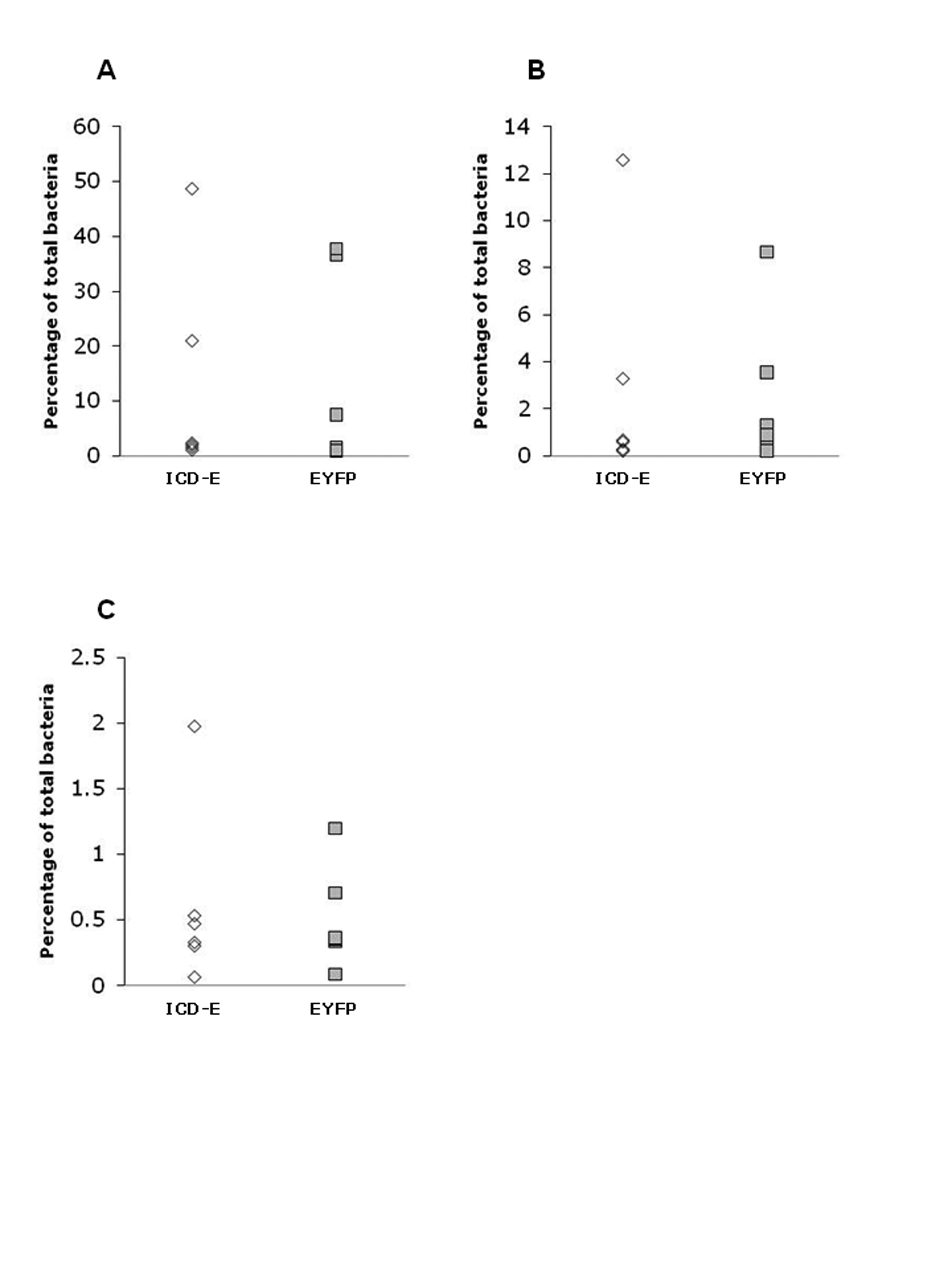

Supplement: Figure S6 — Analysis of fecal DNA. Mice were induced and fecal pellets collected 72 hours later and DNA extracted. Levels of specific groups of bacteria (Bacteroides/Prevotella (A), Lactobacillus/Leuconostoc/Pediococcus (B) and Bifidobacterium (C)) as a proportion of total bacteria DNA, were assessed by qRT-PCR in ICD-E (white) and EYFP (grey) mice. N = 6/genotype. (TIF) [file pone.0020767.s006.tif]
