# Supplementary material for: Intestinal Activation of Notch Signaling Induces Rapid Onset Hepatic Steatosis and Insulin Resistance
Source: PLoS One. 2011 Jun 16;6(6):e20767. doi: 10.1371/journal.pone.0020767 (PMC3116826; doi:10.1371/journal.pone.0020767)
Supplement: Supplementary file 2 [file pone.0020767.s007.doc]

	24h post induction	96h post induction
**Transcripts involved in fatty acid uptake**		
cd36	1.54(+/-0.49)	0.49(+/-0.19)
lpl	0.65(+/-0.34)	0.74(+/-0.41)
ldlr	0.72(+/-0.21)	1.02(+/-0.24)
pparγ	0.84(+/-0.2)	1.08(+/-0.24)
cebpα	1.14(+/-0.38)	0.82(+/-0.25)
alb	0.74(+/-0.52)	0.69(+/-0.45)
**Transcripts involved in free fatty acid esterification**		
lipin1	0.6(+/-0.23)	1.8(+/-1.23)
dgat1	3.33(+/-1.5)	0.69(+/-0.08)
**Transcripts involved in *de novo* lipogenesis**		
srebp	0.95(+/-0.17)	0.81(+/-0.14)
chrebp	0.71(+/-0.23)	1.4(+/-0.29)
acc	0.71(+/-0.22)	1.04(+/-0.29)
scd1	0.53(+/-0.18)	0.93(+/-0.42)
creb1	1.28(+/-0.22)	0.98(+/-0.11)
**Transcripts involved in impaired β-oxidation**		
ppara	0.95(+/-0.21)	0.7(+/-0.13)
**Transcripts involved in impaired triglyceride secretion**		
apob	0.99(+/-0.28)	0.91(+/-0.24)
